# Inferring short tandem repeat variation from paired-end short reads

**DOI:** 10.1093/nar/gkt1313

**Published:** 2013-12-17

**Authors:** Minh Duc Cao, Edward Tasker, Kai Willadsen, Michael Imelfort, Sailaja Vishwanathan, Sridevi Sureshkumar, Sureshkumar Balasubramanian, Mikael Bodén

**Affiliations:** ^1^School of Chemistry and Molecular Biosciences, The University of Queensland, Brisbane, St Lucia QLD 4072, Australia, ^2^Clayton School of Information Technology, Monash University, Clayton, VIC 3800, Australia, ^3^School of Biological Sciences, Monash University, Melbourne, Australia and ^4^Advanced Water Management Centre, The University of Queensland, Queensland, Australia

## Abstract

The advances of high-throughput sequencing offer an unprecedented opportunity to study genetic variation. This is challenged by the difficulty of resolving variant calls in repetitive DNA regions. We present a Bayesian method to estimate repeat-length variation from paired-end sequence read data. The method makes variant calls based on deviations in sequence fragment sizes, allowing the analysis of repeats at lengths of relevance to a range of phenotypes. We demonstrate the method’s ability to detect and quantify changes in repeat lengths from short read genomic sequence data across genotypes. We use the method to estimate repeat variation among 12 strains of *Arabidopsis thaliana* and demonstrate experimentally that our method compares favourably against existing methods. Using this method, we have identified all repeats across the genome, which are likely to be polymorphic. In addition, our predicted polymorphic repeats also included the only known repeat expansion in *A. thaliana*, suggesting an ability to discover potential unstable repeats.

## INTRODUCTION

DNA repeats are ubiquitous in most eukaryotic genomes. Among them, ‘short tandem repeats’ (STRs), or ‘microsatellites’, are repeat sequences that have units between 2 and 6 bp. The repetitive structures of STRs make them highly prone to errors due to slippage during DNA replication and repair, generating new alleles with variable numbers of repeat units. STRs are generally much more polymorphic than other kinds of mutations such as copy number variation and single-nucleotide polymorphisms (SNPs) (1). Because of their high variability, STRs are often used as molecular markers for population analysis, forensic analysis and genealogical DNA testing.

The length variability of STRs is associated with phenotypic variation in many species, with the extremes exemplified by ∼40 genetic disorders in humans (2–9). These disorders are commonly caused by repeat ‘expansion’ where the number of repeat units in a single repeat tract progressively increases during inter-generational transmission and progeny development. Expansions are found predominantly in tri-nucleotide repeats (TNRs), but tetra-, penta- and hexa-nucleotide expansions have also been discovered (10–13). Analysing the variability of STRs is an important step to understand mechanisms that lead to STR instability.

The advances of high-throughput sequencing (HTS) have generated enormous amounts of sequence data at low costs, providing an unprecedented opportunity to study genetic variation. However, making STR variation calls from HTS data is challenging for two main reasons: (i) the amplification of STR loci during sequencing is also subject to slippage, creating copy number errors in read data; and (ii) the low information content of repetitive sequence reads makes it difficult to align them reliably (14–16). Until now, methods have required that at least one read needs to be longer than the full repeat tract, with the ends of the read aligned to the flanks of the repeat. This severely restricts the capacity of methods to characterize repeat lengths bounded by the capability of many current sequencing technologies.

‘Paired-end’ sequencing is increasingly becoming the strategy of choice, in which two (short) reads are sequenced from the two ends of a sequence fragment. In addition to sequence information, paired-end reads provide relative, longer-range positional information to allow for higher specificity in analyses. In this article, we present STRViper, a method that exploits paired-end information for the detection of STR variation from deep sequencing data. STRViper uses Bayesian inference to analyse STR variation by leveraging diverging fragment sizes, and by explicitly recognizing the causes of such. The method only requires that one or more fragments contain the STRs of interest, and hence can be used to study STRs that are longer than the reads produced. Moreover, the Bayesian framework allows the incorporation of other sources of information to support inference.

Here, we show that STRViper can reliably identify STR variation in both simulated and real HTS data. STRViper outperforms the few methods that can be applied to make length-variant calls. Each alternative method falls short of taking advantage of the rich information available in paired-end data that STRViper leverages in a statistically robust manner. In addition, we decipher a large number of loci with repeat variation in the *Arabidopsis thaliana* genome, which can be used for further genetic analyses. Furthermore, with reliable variant calls in hand, we explore the notion of STR ‘variability’. Using this, STRViper predicts the polymorphic repeats across a population of genomes and uncovers several polymorphic repeats including the locus of the only known repeat expansion in *A. thaliana*. We anticipate this ability will enable researchers to suggest candidates for loci at which new unstable repeat sequences can be discovered.

## MATERIALS AND METHODS

### STRViper estimates repeat-length variation

We developed our method on the simple basis that each paired-end sequence fragment will suggest a difference in length between the repeat in the donor genome (from which the fragment originates) and that in the reference genome (to which the two reads are aligned) when the two ends are aligned to the flanking regions of the repeat. To some extent, a difference can be explained by the *a priori* variation in fragment sizes. Our statistical model recognizes this explanation, but as more fragments are observed, it increasingly relies on the tendencies in the data, i.e. the lengths of fragments, when the linked pair is aligned to the reference sequence.

**Figure 1.**
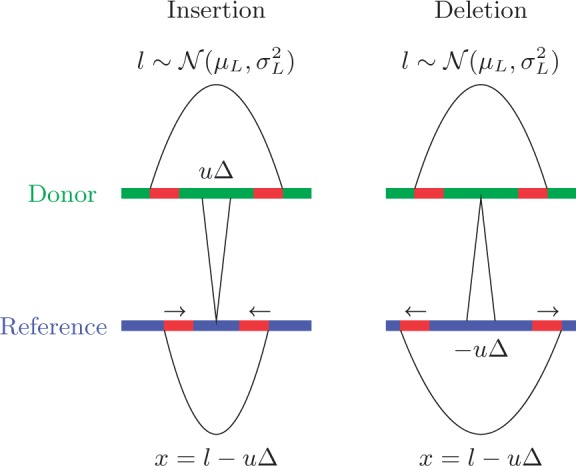
Insertions and deletions cause changes in how reads align to a reference sequence. A fragment with length *l* is sheared from the donor genome and the two ends are sequenced. The linked sequence reads are then mapped to the reference genome. An insertion (left) or a deletion (right) between the two reads in the donor genome will result in an increase or a decrease (respectively) of the observed fragment size *x*.

For a repeat, let 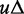 represent a change in length (in nucleotides) relative to a reference genome sequence, where *u* is the repeat unit (e.g. three for TNRs; see Figure 1). We estimate Δ from a set *X* of paired-end fragments with observed (reference sequence) lengths 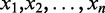, each ‘spanning’ the repeat. More specifically, we place a probability distribution over Δ and use Bayes’ rule to understand how *X* influences the estimate (and the confidence) (see Equation 1).
(1)
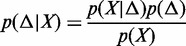



Because fragment sizes are assumed to be normally distributed (and the mean μ and variance 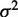 describing the density representing the library are given), observed fragment lengths 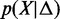 are also normal. We also note that 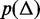 is the length variation known *a priori* to evidence, which can include predictions made by other tools.

STRViper processes sequence data from a SAM/BAM file generated by a read aligner such as BWA (17), Bowtie (18) or Stampy (19). For a given STR, it examines the sizes of specific fragments that span the STR, and the fragment statistics of the library. If the fragment statistics (the mean and standard deviation) of the library is unavailable, the tool will estimate that from all concordant read pairs. STRViper then estimates repeat-length variation by Bayesian inference as described above. The method accounts for the uncertainties of various information sources.

The confidence of variation calls reported by STRViper depends on sequencing depth and the deviation in fragment size. As we demonstrate in Results section, the statistics required for confident calls are practical and within the capacity of current sequencing technologies.

### Details of variation estimation

For an STR locus, let Δ represent the difference in repeat unit number between the STRs in the donor and in the reference genomes. That is, a positive (or negative) Δ indicates an insertion (or deletion) of Δ repeat units in the donor genome. Such an insertion/deletion (indel) causes a change of size 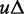, where *u* is the size of a repeat unit. Consider a fragment of size *l* that encompasses the repeat region is amplified. The two reads from the two ends of the fragment are not fully within the repeat region and hence can be reliably mapped to the reference genome. Because of the indel between the two reads, the distance between two ends of the two reads when mapped to the reference genome is 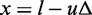 (Figure 1). We refer to this as the ‘observed fragment size’.

Assume a library of paired-end reads is sequenced from the donor genome and the fragment size is normally distributed with mean 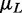 and variance 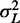. Because of the above linear modification, the observed size of fragments spanning an STR also has a normal distribution with mean 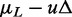 and variance 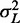, i.e.:
(2)
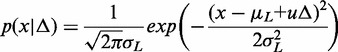



We wish to estimate Δ from a collection *X* of fragments with observed size 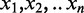 that encompass the STR. To use Bayesian statistics, we place a probability distribution over the variation Δ. We further assume that the prior probability distribution 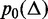 is a normal distribution with mean 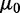 and variance 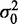 [It will become clear later that this prior probability distribution is a *conjugate prior* of 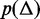]. By applying Bayes’ theorem, we have the posterior probability distribution of Δ given the observed fragment sizes:
(3)
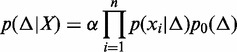

where α is a normalization factor independent of Δ, so that 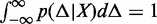. Because 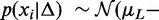 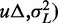 and 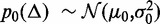, we have:
(4)
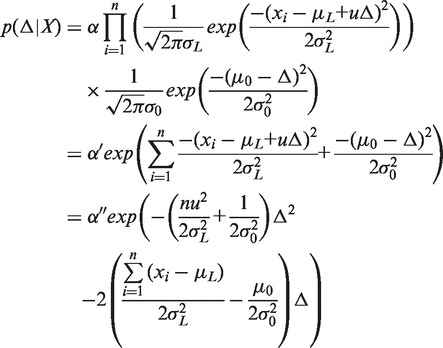

where the constants 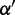 and 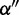 absorb the factors independent of Δ. Here the exponent is a second-order polynomial, which indicates that the posterior distribution 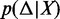 is also a normal distribution with some mean 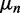 and variance 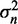.
(5)
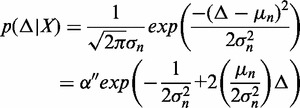



By equating the coefficients in Equations 4 and 5, we obtain:
(6)
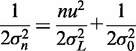



and
(7)
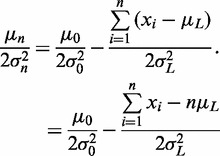



Solving Equations 6 and 7 gives:
(8)
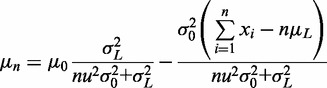



and
(9)
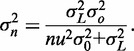



Equations 8 and 9 give an estimate of the variation Δ of an STR by combining information from a prior estimate (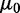 and 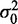) and the observed sizes 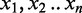 of fragments spanning the STR. Here 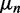 represents the most likely value about Δ and 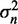 measures its certainty. As 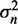 monotonically decreases as *n* increases and 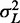 decreases, we can obtain a confident prediction with a large *n* (high coverage) and a small 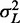 (less variable fragment size).

### Incorporating other sources of information

Bayesian inference allows STRViper to incorporate other sources of information into estimation of variation length. This information is encapsulated in the prior probability distribution 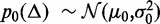
—the prediction of the variation size ‘before’ running this analysis. Such information can be from expert knowledge or other predictions such as that based on intrinsic sequence properties (20) or based on short reads using split reads and read depth coverage signatures. The current STRViper implementation inputs prior predictions in a Variant Call Format (VCF) file, allowing it to be conveniently added to an analysis pipeline with existing tools such as Samtools mpileup (21), Dindel (22) and lobSTR (23). If no prior prediction is available, STRViper uses a bland distribution (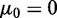 and 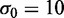) as the prior prediction.

### Simulate STR variation

We first evaluated the performance of STRViper using partially simulated data. We simulated several short read data sets based on the genome of the model organism *A. thaliana*. We obtained the reference accession Columbia (Col-0) genome from release TAIR10 (herein called the reference genome). We then generated three donor genomes, namely, *Sim-1*, *Sim-2* and *Sim-3*, with differing levels of variation by implanting SNPs and short indels into the reference genome. The SNP and indel rates for Sim-2, which are 0.06 and 0.01, respectively, were obtained from the comparative analysis of the *Arabidopsis* strain Bur-0 genome assembly (24) relative to the reference genome. The mutation rates for simulating Sim-1 and Sim-3 were set to 0.5 and 1.5 times that of Sim-2, respectively.

We identified 3685 STRs from the reference genome using TRF (25). For each STR, we computed the variability score (VARscore) using server SERV (20). We scaled the VARscore by a factor and then sampled an STR variation from the obtained value. The scaling factors for simulating STR variation in Sim-1, Sim-2 and Sim-3 were in the ratios 1:2:3. Simulated variation for SNPs, non-STR indels and STRs in the three genomes is described in Table 1.

**Table 1.** Summary of variation in the three synthesized genomes

Genome	Rates		Number Of	Mean STR
	SNP	Indel	Varied STRs	Indel (bp)
Sim-1	0.03	0.005	2257	3.047
Sim-2	0.06	0.010	3079	6.022
Sim-3	0.09	0.015	3199	9.356

Columns 2 and 3 show rates of SNP and indels, column 4 shows the number of varied STRs (of 3685) and column 5 shows the average STR indel size.

We then simulated short reads from the three simulated donor genomes based on Illumina sequencing technology. We generated to 100-folds coverage of paired-end reads with read length of 50 bp and mean fragment size of 200 bp. The error rates for substitutions and indels were set as 0.005 and 0.0005, respectively. To illustrate the performance of STRViper-based fragment size distributions, we generated four read libraries with differing fragment size standard deviation: 10, 15, 20 and 25. These values are consistent with a number of real HTS data sets available such as that reported in (26) (standard deviation of 13) and (24) (between 17 and 25).

### Experimental analysis of STRViper results on real data

We next applied STRViper to a set of real data and experimentally validated its predictions. We obtained read data for 12 *A. thaliana* accessions (Ler-0, Po-0, Ct-1, Sf-2, Rsch-4, Tsu-0, No-0, Hi-0, Edi-0, Wil-2, Oy-0 and Can-0) sequenced by the Wellcome Trust (27) (ENA:ERP000565). Each accession was sequenced with two Illumina paired-end libraries with read lengths 36 and 50 bp, respectively, to between 30 - and 60-fold coverage. The average fragment sizes for the two libraries were 200 and 400 bp. The fragment size standard deviation of these libraries ranged between 7 and 20 bp.

Seeds for 11 *Arabidopsis* accessions (all of the above except for Can-0) were obtained from the European Arabidopsis Stock Centre or Arabidopsis Biological Resource Centre. We used seed stocks N76427 (80 accessions from 1001 genome project), N22660 (96 strains from Nordborg collection), CS76310 (261 strains from Beck and Schaal collection) and the strains sequenced by the Wellcome Trust Centre. DNA from 10-day-old seedlings was extracted and used for polymerase chain reaction (PCR) analysis of the indels predicted by STRViper. We tested 58 TRNs for variability across 11 strains. The primers used for the analysis and additional information are available in Supplementary Table S1. Under standard conditions, PCR was carried out, the fragments were separated on agarose gels and the images were visually inspected to score indels. A couple of representative images representing insertions and deletions are shown in Supplementary Figure S2.

For the computational analyses, we considered an indel of size at least 9 bp as variation. We then compared the variation detected by these tools with the variation observed from gel electrophoresis.

### Analysis of STR variability

The variation of each STR locus for 12 strains was estimated by STRViper. The 12 repeat lengths for the STR from 12 strains, together with the STR length of the reference genome Col-0, made up a sample of 13 lengths. We calculated the unbiased standard deviation of the sample as the variability measure of the STR.

Correlation of genomic properties with STR variability was determined by the Pearson correlation test. The test returned a coefficient indicating the level of correlation between the property and variability, and a *P*-value indicating statistical significance of the observed correlation (the probability of chance explaining it).

Variability of STRs in genomic regions was compared using the Mann–Whitney *U*-test (a.k.a. Wilcoxon rank-sum test). For each region, we compared the variability of STRs placed in such a region against those placed anywhere else. A positive (or negative) U-value indicates that STRs in the particular genomic regions have higher (or lower) variability than those placed in other regions. Again, the *P*-value is the probability that the observed difference in variability distributions can be explained by chance.

### Availability of tool

STRViper is implemented in Java, allowing it to be deployed on most computing platforms. It accepts the standard BAM/SAM format and hence can be easily placed into an existing analysis pipeline. We also provide scripts to parse other data formats such as VCF as well as outputs from Tandem Repeats Finder (25). The tool is freely available at http://bioinf.scmb.uq.edu.au/STRViper.

## RESULTS

### Expected accuracy of repeat-length variation calling

We comprehensively benchmarked STRViper’s performance on controlled, partially simulated data sets, generated under a variety of conditions (see ‘Materials and Methods’ section). We synthesized three genomes, referred to as *Sim-1*, *Sim-2* and *Sim-3*, in increasing order of evolutionary divergence by introducing SNPs and indels into the reference genome accession Col-0 (TAIR10) using mutation rates from a comparative genomics analysis of four diverse *A. thaliana* genomes (24). We further imposed repeat-length variation according to the variability scores provided by SERV (20). Variation statistics is summarized in Table 1.

We simulated the sequencing of the three genomes using Illumina paired-end technology (see ‘Materials and Methods’ section). As the performance of STRViper is dependent on sequencing depth and fragment size distribution, we varied these for each run. Specifically, for each genome, we generated four 100-fold coverage libraries with fragment size standard deviations of 10, 15, 20 and 25 bp, respectively, and applied STRViper to each of them. These standard deviations were observed from analysing short read data sets sequenced by (24) and (27).

We compared the performance of STRViper on these data sets with two existing STR variation detection methods, lobSTR (23) and RepeatSeq (28). We also included two general indel-calling methods, namely, Samtools (21) and Dindel (22). We also ran MoDIL (26), but it yielded poor results in STR variation prediction. MoDIL was not specifically designed for repeat-length variation, and with no available guidance of how to adapt it to work for this problem, we opted not to include it in the comparison. We consistently used Stampy (19) to align reads to the reference genome. Pre-aligned data are required by all tools except lobSTR, which uses its own aligner. For each library, we ran STRViper, lobSTR, RepeatSeq and Samtools at various levels of coverage (10–100-fold, in step of 10-fold) to examine their performance at differing read-depths. (We ran Dindel only on the 10 -, 40 - and 100-fold coverage settings due to its prohibitive computation time.)

We assessed estimates of repeat-length variation using ‘root-mean-squared error’ (RMSE), which measures the difference in sequence units between the real and estimated variation sizes. We also computed the ‘sensitivity’ (the fraction of actual STR variants that are reported) and the ‘positive predictive value’ (the fraction of variant calls that are correct) of the method. We summarized sensitivity and the positive predictive value by the ‘F-score’, which is the harmonic mean between them. A small RMSE and a high F-score are preferred.

**Figure 2.**
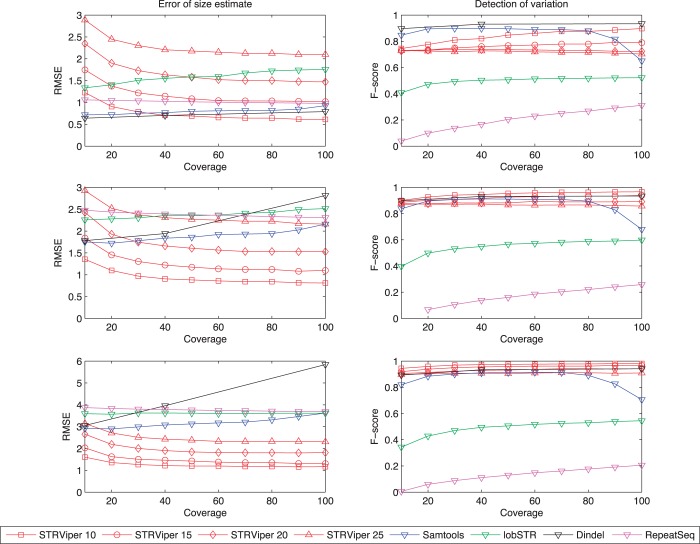
Assessment of repeat-length estimates error (left) and detection of variation call (right) of STRViper, Samtools, lobSTR and Dindel. Top shows performance on Sim-1, middle shows Sim-2 and bottom shows Sim-3. Outcomes are reported for different read depths (coverage). STRViper estimates are provided for fragment size standard deviations of 10–25 nt, as labelled.

The plots in Figure 2 present the RMSE and F-score for the chosen methods on the three genomes. Among these tools, STRViper’s performance largely depends on the fragment size variance, whereas all other methods are indifferent to this. Therefore, we showed STRViper’s performance on four libraries of each genome, while the others’ on one library—the one with fragment size standard deviation of 15. As expected, STRViper’s performance increases (lower RMSE and higher F-score) with tighter fragment size distribution and higher coverage. Most methods increase in accuracy with greater coverage. We note that Samtools’ F-score peaks at coverage of 20–30-fold, and starts to deteriorate when the coverage reaches 70-fold. This is consistent with observations of an earlier review (29).

Sim-1 contains repeat-length variation averaging only 3 bp, at which generic methods Dindel and Samtools are effective, generally more so than STRViper. STRViper requires a tight fragment size library (standard deviation of 10) and high coverage (100-fold) to match their results. Sim-2 contains STR variation ∼6 bp. Here, with fragment sizes with standard deviation <20, STRViper estimates repeat-length variation as well as, or better than Dindel and Samtools, at coverage of ≥40. STRViper has better F-score at lower fragment size deviation. With yet greater repeat-length variation of ∼9 bp in Sim-3, STRViper outperforms other methods at near all settings. Both Dindel and Samtools detect the occurrence of variation in repeat-length well (F-score) for all settings, but fail to estimate the extent of variation (RMSE).

**Figure 3.**
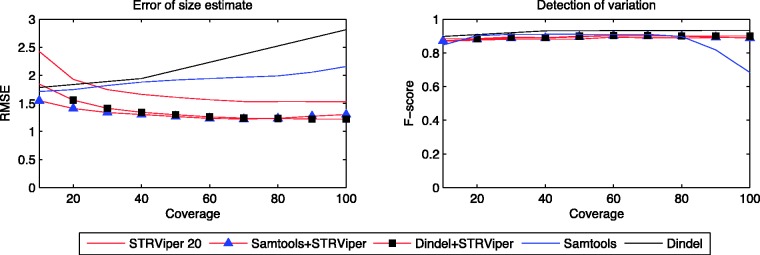
Assessment of repeat-length estimates error (left) and detection of variation call (right) of STRViper when using Dindel and Samtools predictions as prior, and of Dindel and Samtools. Outcomes are reported for different read depths (coverage), on Sim-2 data with fragment size standard deviation 20.

Based on Bayesian inference, STRViper allows other (preferably independent) sources of information to be incorporated. We examined the performance of STRViper when using STR variation calls from Samtools and Dindel as the prior distribution (see Equations 8 and 9). Figure 3 presents the performances of STRViper with the incorporation and compares them with that of Samtools, Dindel and STRViper (with a bland prior). Figure 3 shows the results based on genome Sim-2 with sequence fragment size with standard deviation of 20, a setting where Samtools, Dindel and STRViper perform comparatively. We note that STRViper can leverage the predictions from Samtools and Dindel to improve upon its performance even further.

### Running times

Table 2 presents the average running times of the chosen methods on differing coverage depths. We ran these methods on a cluster of computers, and report here the aggregate CPU times. It is fair to note that Samtools and Dindel seek to locate all indels throughout the donor genomes, while STRViper, lobSTR and RepeatSeq focused only on the 3685 specified STRs. Besides, lobSTR performed its own alignment, whereas STRViper, Samtools and Dindel processed data from a BAM file containing aligned reads. The reported times in Table 2 do not include the alignment times, which were ∼10 CPU hours per 10-fold using Stampy. lobSTR was still the fastest method if mapping times are taken into account, mainly because its mapping algorithm is much more time-efficient than Stampy—lobSTR only attempted to align reads encompassing an STR, while Stampy aligned all reads. Once reads were aligned, RepeatSeq was the fastest. However, both lobSTR and RepeatSeq performances were poor on moderate variation sizes. STRViper needed <4 min to process 10-fold coverage reads, which is 10 and 4000 times faster than Samtools and Dindel, respectively.

**Table 2.** Average running times of STRViper, lobSTR, RepeatSeq, Samtools and Dindel

Method	10-fold	40-fold	100-fold
STRViper	3.86 min	15.50 min	36.50 min
lobSTR	8.70 min	34.52 min	1.55 h
RepeatSeq	0.50 min	1.02 min	1.45 min
Samtools	42.00 min	3.37 h	14.30 h
Dindel	10.88day	46.24day	204.57day

### Comparing STRViper performance with other tools through experimental analysis

To compare the performance of STRViper with other tools on real data, we obtained the raw short-read sequence data from (27) for 12 *A. thaliana* strains (Ler-0, Po-0, Ct-1, Sf-2, Rsch-4, Tsu-0, No-0, Hi-0, Edi-0, Wil-2, Oy-0 and Can-0) and estimated variation for the 3685 STRs found in the *A. thaliana* genomes. These strains were sequenced to 30- and 60-fold coverage, with fragment size standard deviation between 7 and 20 bp (see ‘Materials and Methods’ section). For the purpose of validation, we selected 58 long TNRs (>50 bp) from 11 strains (all above, except for Can-0), amplified the loci through PCR and compared the fragment lengths with that of the reference strain (Col-0) through gel electrophoresis. We scored for each strain whether there is a deletion or insertion or no variation, when compared with Col-0. After removing ambiguous calls from gel image data on the 11 strains, we obtained results for 548 of 638 possible sites (blind to predictions), including 91 deletions and 103 insertions relative to our reference Col-0 (see Supplementary Table S1). We also applied alternative methods (lobSTR, RepeatSeq, Samtools and Dindel) on the same data set, and compared their estimates of variation against the experimental calls. We include the repeat-length variation implied by the original genome analysis of each strain (27).

We observed high agreement between STRViper and the experimental validation (Table 3). STRViper made 142 correct variant calls (75 deletions and 67 insertions) and attained an F-score of 0.742. Owing to the read length limit of the data (only 50 bp), alternative STR variation detection methods performed poorly on these loci. In particular, Samtools, Dindel and lobSTR reported only 38, 36 and 24 correct variants, respectively. RepeatSeq did not report any variant for the 58 loci. Estimates reported by STRViper included all correct calls from lobSTR, all but two of Samtools and all but three of Dindel. The original annotation detected by a hybrid strategy IMR/DENOM (27), which combines iterative read mapping and *de novo* assembly, reported 50 correct variants (F-score = 0.392). These 50 variant calls were recovered by STRViper too. This suggests that STRViper is sensitive, especially on moderately long repeat tracts.

**Table 3.** Detection of experimentally observed STR variation by STRViper and other methods including Gan *et al.*, 2011

Method	TP	TN	FP	FN	F-score
STRViper	142	316	47	43	0.742
lobSTR	24	349	8	167	0.212
RepeatSeq	0	354	0	194	
Samtools	38	344	16	150	0.306
Dindel	36	346	27	139	0.280
Gan *et al.*	50	346	11	141	0.392

TP: number of true positives, TN: number of true negatives, FP: number of false positives and FN: number of false negatives.

We refrain from performing an RMSE-based evaluation, but note that for arbitrarily selected gels, there is strong agreement in estimated levels of variation with those indicated in the gel images (see Supplementary Figure S2).

### Analyses of STR variability

Encouraged by the accuracy of STRViper, we used the variation estimates to analyse the ‘variability’ of STRs in *A. thaliana* genomes. For each STR, we took the sample of the estimated lengths from 13 *A. thaliana* strains—the above 12 strains and the reference genome Col-0 (see ‘Materials and Methods’ section). We then defined variability of a repeat as the ‘standard deviation’ of the sample. We obtained the variability of all 3685 STRs and the subset of 1042 TNRs. The distribution of variability approximately follows the F-distribution (see Supplementary Figure S1). We hypothesize that if a locus is variable, it is more unstable than repeats in general. Consistent with this, we picked up the GAA.TTC repeat in IIL1 gene, which has been found to be expanded in the Bur-0 accession of *Arabidopsis* (6) among the top 10 intronic repeats with highest variability. The variability of the IIL1 repeat is 16, which was in the 94th and 96th percentiles of the STR and TNR sets, respectively. The simple measure of variability thus clearly identifies the only known repeat expansion phenotype in *A. thaliana*. Further, we randomly picked five genes (At1g48400, At1g47300, At1g30270, At1g13270 and At5g03710) that are predicted to be harbouring high variability and analysed their triplet repeats through PCR analysis across 450 strains (see Supplementary Figure S3). Consistent with the predictions from STRViper, we found these genes to be highly polymorphic, indicating that our analysis based on 13 strains can capture the variability at the population level at least for the subset of the tested genomic regions.

Various mechanisms underlying the instability of STRs have been proposed over the past two decades. The direct cause of DNA slippage is thought to be the forming of unusual secondary structures during DNA replication, recombination and repair [see (30)]. Nevertheless, many other factors have been found to be associated with certain unstable STRs, such as transcription and gene expression level (31), distance to the replication origin (32), DNA methylation (33) and histone modifications (34). However, there has been no study to quantify the association of STR instability with these markers, partly due to the limited numbers of known unstable repeats.

Our analyses establish a strong correlation between STR variability and repeat purity, length, CG content of the repeats and of the flanking regions, but not with the distance to the nearest origin of replication (35) (see Table 4). The test results are broadly similar for the STR and TNR sets. We find that STRs in exons and 5′-UTR are significantly less likely to vary, while those in introns and non-functional regions are significantly more variable for both the general group of STRs and TNRs (see Table 5).

**Table 4.** The (Pearson) correlation (ρ) between STR variability and repeat purity, length, CG content, CG content in the flanking regions and distance to the nearest origin of replication

Property	All STRs		All TNRs		
	Ρ	*P*-value	ρ	*P*-value
Purity	0.435	3.2e-170	0.322	1.8e-26
Length	0.231	7.9e-46	0.242	1.9e-15
CG-content repeats	−0.146	6.2e-19	−0.197	1.4e-10
CG-content flanks	−0.170	1.5e-25	−0.161	1.7e-07
Distance to ORC	−0.001	0.94	−0.028	0.36

**Table 5.** Repeat-length variability associated with different genomic regions

Genomic region	All STRs			All TNRs		
	Number	U-value	*P*-value	Number	U-value	*P*-value
Exon	660	−20.40	9.1e-93	463	−10.00	1.1e-23
Intron	425	3.95	7.9e-05	72	2.71	6.6e-03
5′-UTR	356	−6.00	2.0e-09	152	2.36	1.8e-02
3′-UTR	111	−3.76	1.8e-04	45	0.03	9.8e-01
Upstream	510	2.09	3.6e-02	114	1.43	1.5e-01
Downstream	410	1.42	1.5e-01	112	1.93	5.4e-02
OtherRNA	12	−1.45	1.5e-01	8	−1.25	2.1e-01
Non-functional	1525	13.50	1.1e-41	227	5.63	1.8e-08

Absolute counts, Mann–Whitney U- and *P*-values are provided for each genomic annotation.

To investigate whether genes at variable (or non-variable) repeat loci are associated with specific roles, we performed a Gene Ontology enrichment analysis. Specifically, we used the Mann–Whitney *U* test again, to ascertain the difference of variability scores for genes with a particular term *t*, and the scores for genes without *t*. (We test *t* if it is assigned to at least one gene that contains a repeat, either as part of its exons, introns, 5′-UTR or 3′-UTR.) First, we test what terms are associated with genes, with repeats scoring ‘higher’ than expected. As can be seen in Supplementary Table S2, variable repeats are associated with many terms related to a range of metabolic processes. Indirectly, variable loci may explore their roles opportunistically and several of the terms describe traits and processes that are less critical than say developmental ones. It is perhaps more informative to look at roles of genes that do not tolerate (to the same degree) variability of repeat lengths. So, second, we test what terms are associated with genes at repeat loci that vary ‘less’ than expected (see Supplementary Table S3). Here, we see DNA and RNA binding and splicing, nuclear organization including chromatin and development assigned to these non-variable loci.

## DISCUSSION

We have put forward a method that is able to robustly estimate repeat-length variation, and a strategy to identify loci with repeat-length variability. This strategy has highlighted genomic properties that suggest there is a link between the tendency of a repeat to vary in a population of individual (and related) genomes and that of repeat instability. Repeat-length, purity and CG content of the repeats all have an effect on the propensity of the single-stranded DNA to form unusual conformations (36). It is well known that longer repeats and greater repeat purity are more susceptible to instability (37). What we refer to as variable STRs (including, in particular, TNRs) are statistically enriched in all of the above. However, we do not find origins of replication to be associated with the expansion of the tetra-nucleotide repeats (CCTG.CAGG)*_n_* as reported for human (32). Strikingly, we find that IIL1, the only known repeat expansion in *A. thaliana*, is highly variable. We propose that estimates of variation as produced by STRViper can more broadly help discover repeat tracts that are unstable.

We produce and make available estimates of variation for 12 strains of *A. thaliana*, each locus also annotated with the simple variability score we explored (see Supplementary File S1). We verified the predicted indels in 58 TNR repeat tracts of 11 strains sequenced by the Wellcome Trust Centre for Human Genetics (27). Given our accuracy, we now provide a list of 100 indels that are likely to be polymorphic between any two strains (see Supplementary Files S2–S4). Although the use of microsatellite markers is limited, we believe this list would be a valuable addition for some researchers working on genotyping natural variation in *A. thaliana*.

Existing methods for indel detection from HTS data typically use one of three signatures. The ‘depth of coverage’-based approaches used in CNV-seq (38), the pipeline in (39) and BIC-seq (40) assume a uniform coverage of reads across the genome. They expect deletions (or insertions) at a particular location to decrease (or increase) the numbers of reads mapped to that location. These approaches are adversely influenced by the over- or under-sampling caused by the sequencing bias from current technologies. This depth of coverage signature is only significantly strong for large indels (size of ≥50 bp) (41), which is unusual for STR variation. In repetitive regions, reads can commonly be aligned to multiple locations, which further complicates the calculation of coverage.

The second signature for detecting indels is ‘split reads’, which is used in Samtools (21), Dindel (22), lobSTR (23) and RepeatSeq (28). These methods identify variation directly from the differences between a read sequence and the reference sequence, and therefore are sensitive for detection of novel indels but are limited to short STRs due to requiring reads longer than the STR in question. Furthermore, these approaches are dependent on the mapping of reads containing indels, which is often unreliable in repetitive regions.

Methods using the third signature ‘paired-end mapping’ include MoDIL (26), BreakDancer (42) and STRViper. They identify indels from deviant fragment sizes. A key criticism of paired-end mapping approaches is that they require a tight distribution of fragment sizes to make reliable predictions of small indels. However, we have demonstrated that,STR variation can be estimated from realistic fragment size distributions (standard deviation of 20) and sequencing depths (40-fold coverage). STRViper resembles MoDIL in that both assume a normal distribution of fragment sizes and make prediction by comparing this distribution with the distribution of observed fragment sizes. However, the two methods differ in many aspects, notably that STRViper uses full Bayesian statistics that places a distribution over the indel size and allows the incorporation of indel prediction from other methods. STRViper is also specifically designed for STR variation, and hence does not require a computationally expensive clustering to take place to scale well with the large sets of short read data.

Variation can be detected *de novo*, where each genome is assembled separately, and then compared with each other and/or the reference genome. Although this approach reduces the bias from the reference genome, it is computationally expensive. We note that STRViper improves on the *de novo* variant calls published with the original *A. thaliana* sequence data set (27).

Paired-end mapping in general, and STRViper in particular, offers a number of advantages with regard to STR variation detection. First, reads are not required to be longer than the repeat of interest; only the fragments are. Therefore, paired-end mapping analysis is not constrained by current sequencing technologies and can usually be accommodated during library preparation. Second, the two reads from a fragment spanning the STR are not necessarily repetitive, and hence their alignment can be more reliable. As mentioned above, the requirement for fragment size distribution tightness can be compensated by deep sequencing data. Our experiments showed that with a realistic fragment size distribution (standard deviation between 15 and 20) and an achievable coverage depth (40-fold), STRViper performed better than existing methods in detecting STR variation.

The importance of STR instability has been widely recognized because of its association with severe genetic disorders and its use as biological markers. Previous work on analysing STR instability was often limited to measuring variability across species (20) or from a small number of individuals (43). HTS data provided a unique opportunity to study STR variability on a large sample of individuals. However, such analyses (44,45) were limited to short STRs (≤45 bp), while longer STRs are potentially more biologically meaningful. STRViper offers a robust solution to study STRs beyond read lengths, thereby facilitating genome-wide assessment of STR variability. The Bayesian statistics framework also allows STRViper to incorporate sources of information other than paired-end tags to maximize the reliability of results. The method is also computationally efficient, which makes it suitable for analysing such large data sets.

## CONCLUSIONS

We have presented STRViper, a novel and statistically robust method to reliably detect (length) variation in repeats from HTS data. The method makes use of paired-end information, and hence can analyse repeat tracts of sizes beyond the practical read length of current sequencing technologies. Thus, the method enables the study of repeats at sizes of relevance to a range of phenotypes. It was rigorously evaluated favourably against the limited number of tools that can be used for this purpose, both on simulated and real data sets.

We applied the method to identify the repeat-length variation for all STRs in a dozen strains of *A. thaliana*. We performed gel electrophoresis to experimentally test a subset of the repeat loci, and found a high agreement with the variant calls, most which are novel. The method was then applied to delineate repeats that tend to vary across multiple genomes in a population. This notion of variability in a population recovered the only known unstable repeat locus IIL1. Using PCR analysis, we further validated the variability detected from the 12 strains, in 450 strains of *A. thaliana*, covering a diverse group of genomes.

The introduction of STRViper will add an important tool to the analysis of the rich source of data from HTS technologies. It will contribute to the vast applications of studying genome variation alongside the existing techniques for analyses of SNP and large structural variants.

## SUPPLEMENTARY DATA

Supplementary Data are available at NAR Online.

## FUNDING

National Health and Medical Research Council (NHMRC) Project Grant [1004112]; Australian Research Council (ARC) Discovery Project [DP1095325], ARC Australian Postdoctoral Fellowship (to S.S.) [DP110100964] and ARC Future Fellowship [to S.B. FT100100377]; This material is the responsibility of the authors and does not reflect the views of the NHMRC and the ARC. Funding for open access charge: ARC [FT100100377].

*Conflict of interest statement*. None declared.
